# Clinical Efficacy of Probiotics for Allergic Rhinitis: Results of an Exploratory Randomized Controlled Trial

**DOI:** 10.3390/nu16234173

**Published:** 2024-11-30

**Authors:** Lisa Lungaro, Patrizia Malfa, Francesca Manza, Anna Costanzini, Guido Valentini, Diletta Francesca Squarzanti, Elisa Viciani, Alena Velichevskaya, Andrea Castagnetti, Marianna Barbalinardo, Denis Gentili, Alessio Cariani, Sara Ghisellini, Fabio Caputo, Roberto De Giorgio, Giacomo Caio

**Affiliations:** 1 Department of Translational Medicine, University of Ferrara, 44121 Ferrara, Italy; francesca.manza@unife.it (F.M.); anna.costanzini@unife.it (A.C.); guidomaria.valentini@edu.unife.it (G.V.); fabio.caputo@unife.it (F.C.); roberto.degiorgio@unife.it (R.D.G.); 2 SynBalance Srl, 21040 Origgio, Italy; p.malfa@synbalance.care (P.M.); d.squarzanti@synbalance.care (D.F.S.); 3 Wellmicro Srl, 40138 Bologna, Italy; elisa.viciani@wellmicro.com (E.V.); alena.velichevskaia@wellmicro.com (A.V.); andrea.castagnetti@wellmicro.com (A.C.); 4 Institute for the Study of Nanostructured Materials (CNR-ISMN), National Research Council, 40129 Bologna, Italy; marianna.barbalinardo@cnr.it (M.B.); denis.gentili@cnr.it (D.G.); 5 Analysis Laboratory, St. Anna Hospital, 44121 Ferrara, Italy; a.cariani@ospfe.it (A.C.); s.ghisellini@ospfe.it (S.G.); 6 Complex Operational Unit of Internal Medicine, S.S. Annunziata Hospital, Cento, 44121 Ferrara, Italy; 7 Mucosal Immunology and Biology Research Center, Massachusetts General Hospital-Harvard Medical School, Boston, MA 02114, USA

**Keywords:** allergic rhinitis, immune system, inflammation, microbiota, probiotics

## Abstract

**Background:** Allergic Rhinitis (AR) is an atopic disease affecting the upper airways of predisposed subjects exposed to aeroallergens. This study evaluates the effects of a mix of specific probiotics (*L. acidophilus* PBS066, *L. rhamnosus* LRH020, *B. breve* BB077, and *B. longum* subsp. *longum* BLG240) on symptoms and fecal microbiota modulation in subjects with AR. **Methods:** Probiotic effects were evaluated at the beginning (T0), at four and eight weeks of treatment (T1 and T2, respectively), and after four weeks of follow-up from the end of treatment (T3) (*n* = 19) compared to the placebo group (*n* = 22). AR symptoms and quality of life were evaluated by the mini rhinitis quality of life questionnaire (MiniRQLQ) at each time point. Allergic immune response and fecal microbiota compositions were assessed at T0, T2, and T3. The study was registered on Clinical-Trial.gov (NCT05344352). **Results:** The probiotic group showed significant improvement in the MiniRQLQ score at T1, T2, and T3 vs. T0 (*p* < 0.01, *p* < 0.05, *p* < 0.01, respectively). At T2, the probiotic group showed an increase in *Dorea*, which can be negatively associated with allergic diseases, and *Fusicatenibacter*, an intestinal bacterial genus with anti-inflammatory properties (*p*-value FDR-corrected = 0.0074 and 0.013, respectively). Conversely, at T3 the placebo group showed an increase in *Bacteroides* and *Ruminococcaceae unassigned*, (*p*-value FDR-corrected = 0.033 and 0.023, respectively) which can be associated with allergies, while the probiotic group showed a significative increase in the *Prevotella*/*Bacteroides* ratio (*p*-value FDR-corrected = 0.023). **Conclusions:** This probiotic formulation improves symptoms and quality of life in subjects with AR, promoting a shift towards anti-inflammatory and anti-allergic bacterial species in the intestinal microbiota.

## 1. Introduction

Allergic rhinitis (AR) is an atopic disease affecting the upper airways, occurring when predisposed subjects are exposed to aeroallergens, such as pollens, dust mites, and animal dander. AR is caused by an immunoglobulin E (IgE)-mediated type 1 hypersensitivity response and is characterized by nasal congestion, watery rhinorrhea, sneezing, postnasal drip, and nasal, eye, and throat itching. Depending on the duration of the symptoms, AR is classified as intermittent, if symptoms last for less than 4 days per week or less than 4 consecutive weeks, and persistent, if symptoms last for more than 4 days per week for at least 1 month [1]. Patients affected by AR complain of tiredness, fatigue, and reduced quality of life (QoL). Indeed, AR comes with heavy social costs by reducing work productivity, impairing education, and raising the costs of the healthcare system due to disabling symptoms. AR management primarily relies on: (i) avoiding the allergens that cause the reaction; (ii) nasal rinsing; (iii) pharmacological treatment, and (iv) allergen-specific immunotherapy. The pharmacological management of AR includes intranasal and oral antihistamines to improve histamine-triggered symptoms and nasal corticosteroids to ameliorate nasal obstruction. On the other hand, allergen-specific immunotherapy is still the only causal treatment. Also, some nutraceuticals have been investigated for symptom relief [2,3,4]. AR symptoms result from immune mediators released in nasal tissue during an allergic reaction. The pathogenetic mechanism of AR begins with sensitization, i.e., the initial exposure of an individual to an allergen without the presence of clinical symptoms. During this phase, dendritic cells in the nasal mucosa process and transport the allergen to the draining lymph node, presenting it to naive CD4+ T cells. These T cells differentiate into allergen-specific type 2 T helper (TH2 cells), leading to the activation of B cells and their transformation into plasma cells that produce allergen-specific IgE. The IgE circulate in the blood stream and bind to the high-affinity IgE receptor (FcεRI) on effector cells, such as mast cells and basophils, creating a pool of memory allergen-specific TH2 cells and B cells [4,5]. In a previously sensitized individual, re-exposure to the same allergen triggers the binding of allergen-specific IgE on mast cells in the nasal mucosa, causing crosslinking of IgE and FcεRI. This leads to mast cell activation and degranulation, releasing a wide array of pre-stored and newly synthesized mediators, e.g., histamine, sulfidopeptide leukotrienes (leukotriene C4 and leukotriene D4), prostaglandin D2, and other inflammatory cytokines. These mediators interact with nasal sensory nerves, vessels, and glands, resulting in acute AR symptoms [4].

Based on the evidence that more than 70% of the cells of the immune system are located in the gut [6] and that the microbiota plays a leading role in modulating the inflammatory process and the immune response, probiotics have recently been considered a new treatment option also for many diseases including AR [5,7,8]. The Food and Agriculture Organization (FAO) and the World Health Organization (WHO) define probiotics as “live microorganisms which, when administered in adequate amounts, confer a health benefit on the host” [9]. Indeed, the gut microbiota orchestrates various essential physiological activities, including protective, metabolic, trophic, and immune functions. Probiotics aid in food digestion by fermenting partially digested and indigestible polysaccharides, resulting in the production of short-chain fatty acids (SCFAs) such as acetate, propionate, and butyrate. SCFAs directly regulate intestinal immune homeostasis by promoting T-cell polarization and differentiation into effector and regulatory T cells (Tregs) [10,11]. Furthermore, SCFAs help maintain the proper functioning of the intestinal barrier [12,13,14,15] and enhance pathogen-specific secretory IgA (sIgA) responses without affecting probiotic-specific sIgA [16,17]. Additionally, SCFAs prevent the apoptosis of epithelial cells, contributing further to intestinal barrier integrity [18,19]. Also, probiotics contribute to the host’s health by producing post-biotics (e.g., vitamins and neurotransmitters), which can modulate the immune, endocrine, and central nervous systems while providing a protective effect against pathogen colonization.

It is now well understood that changes in the normal richness and diversity of gut microbiota (i.e., dysbiosis) lead to the disruption of tight junctions. As a result, intestinal mucosa permeability increases, triggering an inflammatory cascade and subsequent cytokine production [20]. Scientific evidence indicates that probiotics can play a crucial role in restoring the gut microbiota eubiosis and producing antibacterial molecules (e.g., bacteriocins, SCFA, organic acids, hydrogen peroxide) that can directly kill or inhibit pathogens [21]. Furthermore, probiotics are known to protect against pathogens by the following methods: (a) reducing the expression of virulence factors necessary for colonization; (b) disrupting pathogen’s cell walls; (c) reducing their mobility and viability; and (d) enhancing the host’s ability to eliminate intracellular pathogens [22,23,24,25].

Further evidence indicates that probiotics exert anti-allergic properties and influence respiratory allergies through the gut-lung axis by modulating intestinal dysbiosis, an event ensuing immune tolerance toward allergens. Probiotics exert immunomodulatory effects through various mechanisms: (i) increasing the Th1/Th2 ratio, which stimulates Th1-mediated cytokine production and reduces Th2-mediated cytokine production; (ii) decreasing the infiltration of lymphocytes and eosinophils in the respiratory tract, reducing the production of allergen-specific IgE, and increasing levels of allergen-specific IgG1 and IgG2; and (iii) elevating butyric acid levels, while enhancing IgA production, and (iv) promoting the production of anti-inflammatory cytokines and reducing the level of pro-inflammatory molecules [22,26,27,28]. For this reason, over the past decade, probiotics have been effectively used in numerous clinical studies addressing immune system abnormalities, such as autoimmune diseases, inflammatory conditions, atopic dermatitis, and respiratory tract infections [29,30,31,32]. The extensive literature on probiotics highlights the importance of strain specificity and the need for studies that demonstrate their actual benefit [33].

This study aimed to evaluate the effect of selected probiotic strains (*Lactobacillus acidophilus PBS066*, *Lacticaseibacillus rhamnosus* LRH020, *Bifidobacterium breve* BB077, and *Bifidobacterium longum* subsp. *longum* BLG240) for the treatment of AR. The probiotic strains evaluated in this study have already shown antimicrobial efficacy in vitro and significant anti-inflammatory activity both individually and in combination [34,35]. They have demonstrated the ability to adhere to the intestinal epithelium, increase antioxidant potential, optimize immune response, and positively modulate intestinal biodiversity [36]. Additionally, these strains were evaluated in a clinical study focused on constipation in irritable bowel syndrome, demonstrating their ability to colonize and persist in the gut, reducing symptoms both in the short- and long-term supplementation [37].

## 2. Materials and Methods

This randomized, double-blind, placebo-controlled, parallel-group clinical study was conducted at St. Anna University Hospital, Cona (Ferrara), Italy, between March and July 2022. With a 5% margin of error and 80% confidence level, a maximum of 43 patients would be required to represent the proportion of the Italian population that is aged 18–60 and diagnosed with AR. A total of 44 patients suffering from persistent or seasonal AR (aged 18–60 years) were recruited and screened. One of these, a female patient, did not satisfy the inclusion criteria, thus she was excluded from the study. Forty-three patients were randomized in placebo (Placebo, *n* = 22) or probiotic group (Probiotic, *n* = 21). Forty-one of the 43 enrolled subjects completed the study. Two from the Probiotic group discontinued the trial for personal reasons. Therefore, the final number of patients who received the probiotics was 19. The study was approved by the Ethics Committee Area Vasta Emilia Centro of the Emilia-Romagna Region (CE-AVEC) number 678/2021/Sper/AOUFe and registered on Clinical-Trial.gov (NCT05344352) on 19 April 2022. All the patients signed the informed consent.

Inclusion and exclusion criteria applied to healthy subjects of both sexes are listed in Supplementary Table S1.

### 2.1. Study Product

The probiotic formulation used in the study, containing *L. acidophilus* PBS066 (DSM24936), *L. rhamnosus* LRH020 (DSM25568), *B. breve* BB077 (LMG P-30157) and *B. longum* BLG240 (LMG P-29511), and the placebo product were provided by Synbalance Srl (Origgio, VA, Italy). All the products were produced according to the good manufacturing practices in a certified manufacturing facility. The probiotic food supplement (Probiotic) was formulated in capsules of hydroxypropylmethylcellulose and pectin containing *L. acidophilus* PBS066 1 × 10^9^ UFC, *L. rhamnosus* LRH020 1 × 10^9^ UFC, *B. breve* BB077 1 × 10^9^ UFC, and *B. longum* BLG240 1 × 10^9^ UFC plus excipients (maltodextrin, corn starch, and magnesium salt of fatty acids). The placebo was formulated in identical capsules and contained only bulking agents.

### 2.2. Study Design

**Figure 1:**
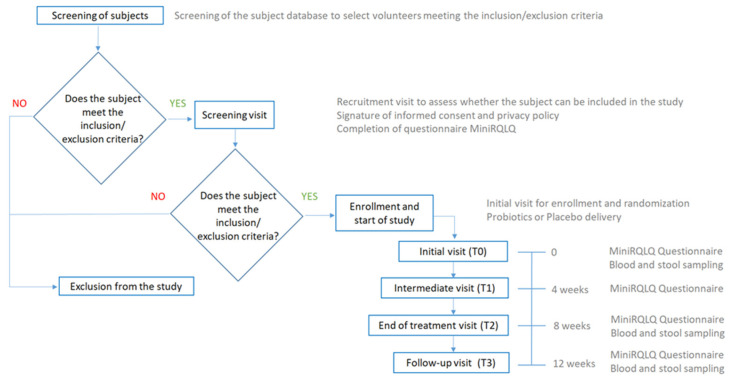
Study flow-chart.

The Placebo group was administered with a product containing only bulking agents normally used in the formulation of food supplements. The Probiotic group was treated with the combination of probiotic strains (*L. acidophilus* PBS066, *L. rhamnosus* LRH020, *B. breve* BB077, and *B. longum* BLG240) added with bulking agents. Participants swallowed one capsule containing the probiotic combination or placebo daily, separate from meals, for eight weeks. The effects of the treatment were evaluated at the beginning (T0), at four (T1) and eight weeks (T2) of treatment, and after four weeks of follow-up from the end of the treatment (T3, total period: twelve weeks). The allergic immune response was assessed by inflammatory blood markers (i.e., eosinophil count, eosinophil cationic protein [ECP], and total immunoglobulin E [IgE]), at T0, T2, and T3, respectively. AR symptoms and QoL were evaluated by the Mini Rhinoconjunctivitis Quality of Life questionnaire (MiniRQLQ) at each time point. Fecal microbiome profiling was determined by 16S rRNA gene sequencing analysis at T0, T2, and T3. Figure 1 describes the study flowchart.

### 2.3. Questionnaire

#### Mini Rhinoconjunctivitis Quality of Life Questionnaire (MiniRQLQ)

The Mini Rhinoconjunctivitis Quality of Life questionnaire (MiniRQLQ) is a 28-question questionnaire assessing the QoL. The questionnaire investigates the AR impact on regular activities (e.g., work, social activities), sleep, emotional aspects, with a scale ranging from 0 (no discomfort) to 6 (very annoying). The higher the total score, the worse the conditions. Minimal clinically important differences were defined as a reduction  ≥0.4 point for the mini RQLQ, according to Barnes et al., 2010 [38].

### 2.4. Measurement of Inflammatory Blood Markers

#### 2.4.1. Blood Eosinophil Count

The blood eosinophil count was performed with the flow cytometric method on Dasit’s XN 1000 analytical platform (Sysmex, Lincolnshire, IL, USA).

#### 2.4.2. Blood Cationic Protein of Eosinophils (ECP)

The measurement of the blood cationic protein of eosinophils was carried out with the FEIA (Fluoroenzymeimmunoassay) method on the Thermofisher Immunocap 1000 platform with proprietary ImmunoCAP™ ECP kit, (Thermofisher Scientific, Milan, Italy).

#### 2.4.3. Total Immunoglobulin E

The Total serum IgE level were determined accordingly with “ECP- ImmunoCAP™ Total IgE kit Measurement” (Thermofisher Scientific, Milan, Italy).

### 2.5. Next Generation Sequencing

#### 2.5.1. DNA Extraction and Purification

Total microbial DNA was isolated from fecal samples utilizing the DNeasy 96 PowerSoil Pro QIAcube HT Kit on the QIAcube HT instrument (QIAGEN, Hilden, Germany), adhering to the manufacturer’s guidelines. Prior to the total DNA extraction, a bead-beating process was conducted using Lysing Matrix E (MP Biomedicals) on a FastPrep24 bead-beater (MP Biomedicals, Irvine, CA, USA) at a rate of 6.0 movements per second for 40 s. Negative controls consisted of PCR-grade water, which underwent the same library preparation steps and Next Generation Sequencing (NGS) as the other samples. DNA quantification was performed using the Qubit™ 4 Fluorometer (Fisher Scientific Italia, Segrate, Italy).

#### 2.5.2. Determination of Bacterial Profiles by Amplicon Sequencing

The amplification of the V3 to V4 region of the 16S rRNA gene was accomplished using the specific primer set S-D-Bact-0341-b-S-17/S-D-Bact-0785-a-A-21 [39]. To prepare indexed libraries, limited-cycle PCR with Nextera technology (Illumina, San Diego, CA, USA) was employed, followed by cleanup using VAHTS DNA Clean Beads (Vazyme, Red Maple Hi-tech Industry Park, Nanjing, China). The resulting libraries were pooled at equimolar concentrations of 4 nM, denatured, and subsequently diluted to 5 pM for sequencing. The sequencing was conducted on the MiSeq platform using the MiSeq Reagent kit V3 (Illumina, San Diego, CA, USA), following a 2 × 300 bp paired-end protocol as per the manufacturer’s guidelines. The raw sequence data generated from this process has been archived in the Sequence Read Archive (SRA) under the project number PRJNA1164129.

#### 2.5.3. Data Processing and Analysis

Patients who failed to provide samples either at the beginning or end of the study were excluded from the analysis. The sequenced reads of the patients’ gut microbiota were assessed using QIIME2 (version 2020.6) [40]. The DADA2 (Divisive Amplicon Denoising Algorithm 2) plugin facilitated the removal of noise and chimeric sequences, leading to the generation of Amplicon Sequence Variants (ASVs) [41]. VSEARCH 2020.6.0 was utilized for quality filtering and clustering [42]. High-quality reads were taxonomically classified using the SILVA reference database version 132 [43]. The taxonomic classification of each Lactobacillus ASV was validated by aligning the ASV sequences with the NCBI Nucleotide Collection (nr/nt), specifically against the Lactobacillus database (tax id: 1578), by the online Nucleotide BLAST program [44]. The data were imported into R (version 4.2.2) [45] via RStudio Team 2020 [46] where subsequent analyses were conducted using the R packages phyloseq, rbiom, ggplot2, tidyverse, tidyr, vegan, ape, ggpubr, and dplyr [47,48,49,50,51,52,53,54]. Environmental microbial contaminants were eliminated from the analysis by filtering out ASVs that were included in the negative controls, applying the decontam R package at a 5% stringency [55]. Normalization by rarefaction without replacement was executed to adjust for the varying sequencing depths of each sample, standardizing to 10,569 reads, at which point the samples achieved the maximum total ASV number asymptote. Differences in alpha diversity were assessed based on the distribution of metrics, using ANOVA and Tukey’s HSD (honestly significant difference) tests for normally distributed data, or the Wilcoxon–Mann–Whitney test with Holm–Bonferroni correction (WMW with HB) for non-normally distributed data. To compare microbial composition across samples, beta diversity was quantified by calculating both weighted and unweighted UniFrac distance matrices based on bacterial data [56]. Principal coordinates analysis (PCoA) was performed on these distance matrices to create bi-dimensional plots in R. The dispersion of the PCoA clusters was determined using the betadisper function from the R vegan package [57]. A permutational analysis of variance (PERMANOVA) test calculated via the adonis2 function in the vegan package was conducted to assess whether there was a significant separation between various sample groups. The linear discriminant analysis (LDA) effect size (LEfSe) algorithm, accessible through the Galaxy web application at https://huttenhower.sph.harvard.edu/galaxy/ (accessed on 22 December 2023), was applied to identify bacterial taxa correlated with the treatment [58]. Differences in abundance were deemed significant when the logarithmic LDA score exceeded 2.

### 2.6. Statistics

#### 2.6.1. Questionnaires

Data are reported as mean ± SE (standard error). The intragroup statistical analysis (vs. baseline values) is reported as follows: * *p* < 0.05, ** *p* < 0.01, *** *p* < 0.001. The intergroup statistical analysis (Probiotic vs. Placebo) is reported as follows: # *p* < 0.05, ## *p* < 0.01, ### *p* < 0.001. The Statistical test applied is the one-tailed Student’s *t* test.

#### 2.6.2. NGS Statistical Analysis

A permutational multivariate analysis of variance (PERMANOVA, utilizing 999 permutations) was conducted to assess differences in microbial beta diversity among groups. Differentially abundant taxa were determined using linear discriminant analysis (LDA) effect size (LEfSe). Categorical variables are reported as counts and percentages while continuous variables are shown as median, minimum, and maximum values. To compare groups, normality of the data were checked using either the Shapiro–Wilk test or the Kolmogorov–Smirnov Test [59]. Fisher’s exact test [60] was applied for categorical variables, the Mann–Whitney U test [61] was used to non-normally distributed continuous data, and the *t*-test [62] was used for normally distributed continuous data.

## 3. Results

### 3.1. Questionnaire

**Figure 2:**
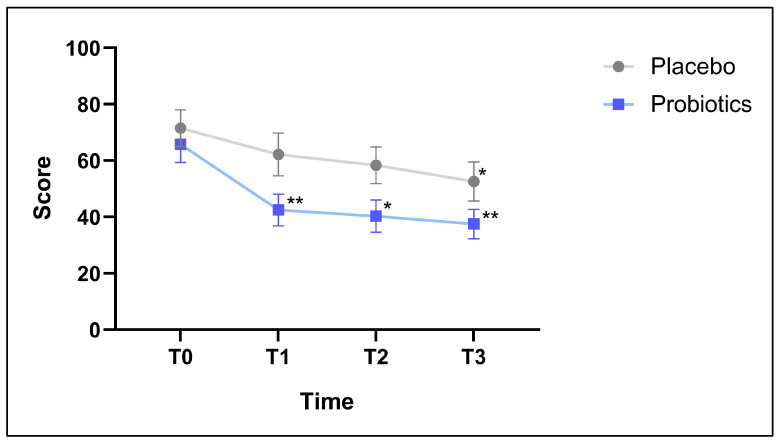
Mini Rhinoconjunctivitis Quality of Life questionnaire (MiniRQLQ). The asterisks indicate comparison between initial (T0) and T1, T2, T3 data within the same group (Placebo group, *n* = 22 and Probiotic group, *n* = 19). * *p* < 0.05, ** *p* < 0.01.

In MiniRQLQ questionnaires, the higher the score, the worse the QoL condition was. The Placebo group reported a significant improvement in their health at T3 compared to their baseline values (intragroup variation), while the group treated with the combination of probiotic strains reported a significant intragroup variation at T1, T2, and T3 vs. T0 (*p* < 0.01, *p* < 0.05, *p* < 0.01, respectively) (Figure 2).

### 3.2. Gut Microbiome Characterization in Probiotic and Placebo Groups

Gut microbiota sequencing on the patient fecal samples was unsuccessful on two Placebo and on two Probiotic subjects. Thus, their respective time points had to be dropped from the final NGS analysis. Twenty Placebo subjects and seventeen Probiotic subjects were then analyzed.

**Figure 3:**
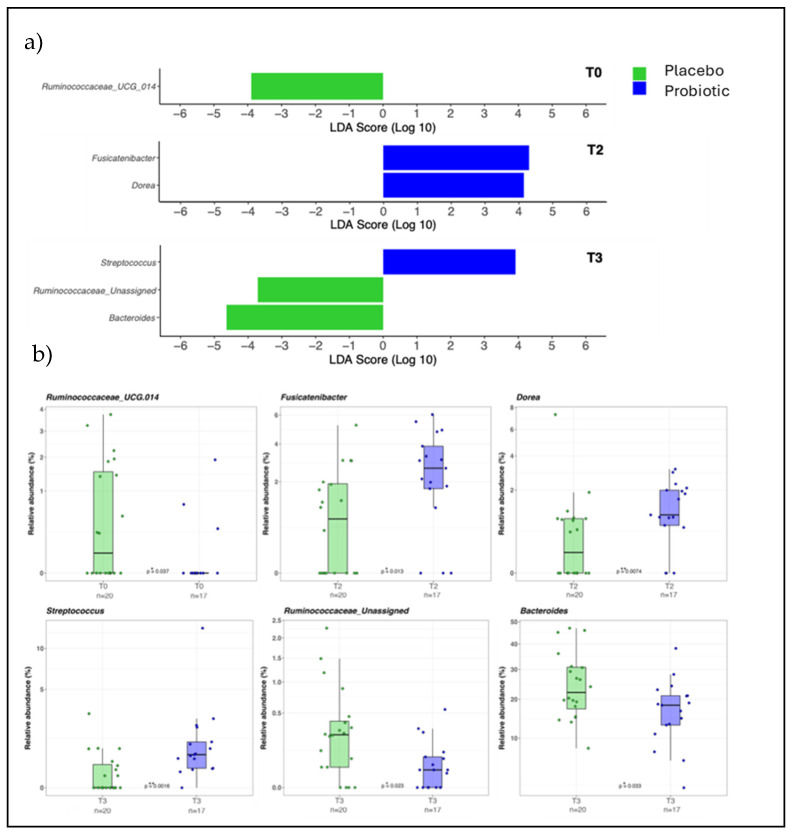
(**a**) Plot from LDA LEfSe analysis and (**b**) taxa relative abundances (%). The length of the bar columns represents the LDA score. Box-and-whisker plots with data points are visualized in the six panels at the bottom of the figure and show the relative abundance of each taxon indicated in the LDA LEfSe plots in the Placebo (*n* = 20, lime green) and Probiotic (*n* = 17, blue) groups. Median, first, and third quartiles are shown in the box-and-whisker plots. Mann–Whitney U Test results of the group comparisons are shown. * *p*-value FDR-corrected < 0.05, ** *p*-value FDR-corrected < 0.01. Placebo group, *n* = 20 and Probiotic group, *n* = 17.

**Figure 4:**
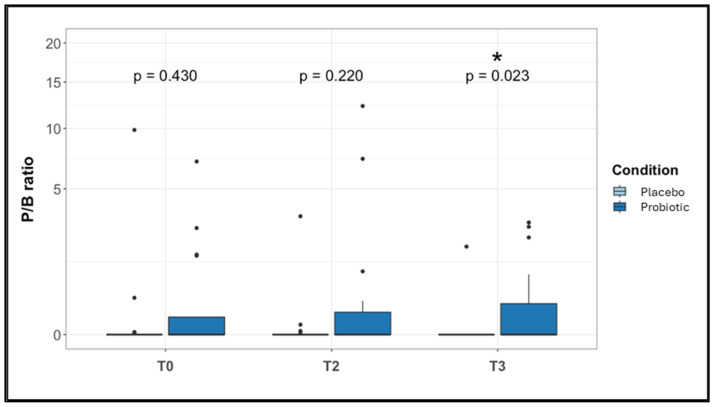
*Prevotella*/*Bacteroides* ratio in the Placebo and Probiotic groups across the three time points of the study. Mann–Whitney *U* Test results of the group comparisons are shown. * *p*-value FDR-corrected < 0.05.

The alpha diversity indexes did not show significant changes between the two study groups at each time point considered, as well as the beta diversity which was evaluated using the UniFrac unweighted and weighted distance metric (Supplementary Figures S1 and S2). The proportions of phyla were maintained at each time point within the study groups. *Bacteroidetes* and *Firmicutes* were the most represented phyla across all the samples (Supplementary Figure S3). The LDA LEfSe algorithm was applied on the dataset to search for the presence of bacterial biomarkers of the two different conditions at the three experimental time points. At the beginning of the study (T0), the Placebo subjects were positively associated with the *Ruminococcaceae UCG*-*014* genus (Figure 3a,b; *p*-value FDR-corrected = 0.037). However, this feature was not maintained by the Placebo group across the three time points. At T2, the relative abundance of *Dorea* and *Fusicatenibacter* was higher in the Probiotic group than in subjects receiving placebo (Figure 3a,b; *p*-value FDR-corrected = 0.0074 and 0.013, respectively). At T3 (i.e., after one month without treatment), the Placebo group showed a positive association and a higher relative abundance of *Bacteroides* (Figure 3a,b; *p*-value FDR-corrected = 0.033), and *Ruminococcaceae unassigned* (Figure 3a,b; *p*-value FDR-corrected = 0.023) compared to the Probiotic group that showed instead a positive association and a higher abundance of *Streptococcus* with respect to the Placebo group (Figure 3a,b, *p*-value FDR-corrected = 0.016). At T3, the Probiotic group showed also a significative increase in the *Prevotella*/*Bacteroides* ratio (*p*-value FDR-corrected = 0.023) (Figure 4).

## 4. Discussion

AR is a chronic inflammation of the nasal mucosa associated with symptoms (e.g., stuffy and/or runny nose, sneezing, dry eyes) that impairs QoL of affected patients. Probiotics are live bacteria with proven immunomodulatory properties which are intensively studied in a wide range of clinical conditions, from inflammatory bowel diseases to postpartum depression and autism spectrum disorder [29,63,64]. Experimental studies have shown that probiotics have strain-specific effects within the intestinal lumen and on epithelial and immune cells, exhibiting anti-allergic potential. These effects encompass improvements in antigen degradation and gut barrier function, as well as the induction of regulatory and pro-inflammatory immune responses, the latter generally occurring outside the intestinal epithelium [65]. Various strains of Lactobacillus spp. are recognized for their significant role in mediating immune responses through the presence of specific suppressive DNA motifs that are involved in stimulating immunity [66,67]. During allergic reactions, the activation of TLR-9 receptors can disrupt the immune response by inhibiting Th1 cells, thereby potentially counteracting the Th2-type immune response associated with allergies. Immunosuppressive motifs can inhibit and deactivate dendritic cells, while also promoting the conversion of Tregs, which are crucial for triggering allergic cascades [66,67]. Additionally, the probiotic bioactive compound D-tryptophan has been shown to suppress the development of allergic airway disease by limiting the production of Th2 cells and chemokines and enhancing gut microbial diversity [68]. Notably, the biological mechanism of tryptophan has been linked to allergy through its involvement in the degradation process of the immune-regulatory enzyme indoleamine 2,3-dioxygenase (IDO-1), a process mediated by IFN-γ [69]. This is particularly significant, as IFN-γ is a potent mediator of IDO-1, which subsequently degrades essential amino acids as part of an immunoregulatory pathway aimed at preventing excessive immune system activation.

The aim of this study was to evaluate the effects of a combination of specific probiotic strains on symptoms and fecal microbiome of patients with AR.

The MiniRQLQ questionnaire did not show statistically intergroup differences; however, it described a significant improvement in the QoL inside both groups (Placebo and Probiotic) over time. The general improvement of the overall QoL observed in both groups at the end of follow-up period could be explained with the natural progression of the summer season, notoriously less endowed in pollen than the spring season in our geographical area [70]. In particular, the intragroup analysis of the Probiotic group reveals a significant QoL improvement between the start and the first month of treatment (T0 vs. T1), the end of the treatment (T0 vs. T2) and the end of follow-up (T0 vs. T3). Conversely, the only significant difference observed in the intragroup analysis of the Placebo group was between the beginning of the treatment and the end of the follow-up (T0 vs. T3). Our finding, showing that this probiotic formulation is able to improve the QoL of patients with AR after four, eight, and twelve weeks from the starting of treatment respect to baseline (T0), is in line with a previous clinical study, demonstrating the improvement of the global MiniRQLQ score in patients with seasonal allergies who were administered the probiotic strains of *Lactobacillus gasseri* KS-13, *Bifidobacterium bifidum* G9-1, and *B*. *longum* MM-2 [71] for 8 weeks. These results were confirmed by a meta-analysis evaluating 28 studies assessing the effect of probiotics on AR symptoms and reporting an improvement in the Rhinoconjunctivitis Quality of Life Questionnaire (RQLQ) score in the Probiotic group vs. Placebo (SMD, −0.64, 95% CI [−0.79, −0.49], *p* < 0.00001, I2 = 97%) [72]. The probiotic associated improvement of QoL in AR patients is extremely important as symptoms affect sleep, reduce school and work performance and, in the long term, can lead to the development of anxiety and depression [4,73,74,75].

Blood test results showed that this probiotic complex did not improve the inflammatory markers, i.e., eosinophil count, ECP, and tIgE, over time (Supplementary Figure S4, *p* > 0.05). The lack of significant differences in blood eosinophil levels between the two groups is consistent with the results of a meta-analysis by Yan et al., evaluating the effect of probiotic treatment on 2708 subjects with AR enrolled in 30 RCTs. The analysis highlighted no significant difference in blood eosinophil count, despite nasal symptom improvement [18]. IgE values did not decrease in the Probiotic group. This finding is in line with Yan et al. and another meta-analysis by Luo et al. reporting the absence of significant differences in the values of total IgE and specific IgE between AR patients treated with probiotics vs. placebo. The two meta-analyses suggested that the improvement in the clinical outcome, in particular QoL, of probiotics treated AR patients may not depend on IgE levels and eosinophil count, rather it can be determined by mechanisms modulated by probiotics, which influence the human immune mechanisms not yet identified [18,72].

Although routinely used in allergic trials, these markers (i.e., eosinophil count, ECP, and tIgE) may be not adequate for evaluating the response to probiotic treatment [71,76,77]. Therefore, other immune-system actors may play a role in the immunomodulatory mechanisms by which probiotics ameliorate the clinical condition, i.e., the Th1/Th2 ratio and the allergen-specific IgG4. This possibility is supported by findings from the meta-analysis by Luo et al. that included 28 studies, 4 of which evaluated the Th1/Th2 ratio, and reported an increase in this ratio in the Probiotic group vs. the Placebo group. Thus, probiotics may favor the re-balancing of the Th1/Th2 ratio, allowing for symptom improvement in AR [72].

A study evaluating the synergistic effect of *C. butyricum* and Allergen Immunotherapy (AIT) reported a more significant reduction in symptoms and a higher production of allergen-specific IgG4 in subjects treated with the combined therapy, compared to AIT alone, after six months of treatment [78]. In AIT, the immunological mechanism leading to symptom amelioration relied on the shift from IgE to allergen-specific IgG4 production. As a result, Th2 lymphocytes decrease, whereas Tregs increase and the latter effect is associated with IL-10 release resulting in allergen-specific IgG4, which binds to the allergen, thereby blocking the IgE mediated effects [73] including mast cell activation [79]. IgG4 is a biomarker of AIT, and its serum levels correlate with the patient’s clinical response [4,73]. One may speculate that also the probiotic formulation of this study improved the clinical picture of AR by immunomodulating the Th1/Th2 ratio and stimulating the production of allergen-specific IgG4. Further studies evaluating the effect of probiotics on these two immune markers in patients with AR are eagerly awaited.

In the Probiotic group, the ECP value did not decrease throughout the study period. However, several considerations should be raised about ECP. This protein is released by eosinophils in the late phase of the allergic hypersensitivity reaction, although only in a subset of all allergic subjects [4,77].

With regard to IgE, it must be taken into consideration that the allergic inflammatory process co-existed with a non-IgE-mediated inflammatory mechanism triggered by the protease action of allergens that damage epithelial cells and increase the permeability of the intestinal epithelial barrier. This, in turn, evokes a Th2-mediated inflammatory response and the release of cytokines and chemokines. Furthermore, total IgE has a poor predictive value for the screening of AR and is not used in the diagnosis. Specific IgE is used in the diagnosis, but levels of specific IgE generally did not correlate with the severity of symptoms [77]. Hence, using serum IgE values as the only marker of clinical response remains controversial. There are subjects who produce IgE in response to allergens, but they do not have symptoms. Furthermore, some subjects present an abnormal concentration of IgE in tissues and at the mucosal level rather than in the bloodstream [71]. Therefore, it could be hypothesized that these blood markers, which are commonly used to observe changes in the immune response of AR after probiotic treatment, may not be fully reliable.

The results of fecal microbiota analysis reveal no significant change in alpha diversity indexes and beta diversity between the two study groups in all the analyzed time points (T0, T2, and T3). These findings are consistent with previous data reporting that the treatment with probiotics in AR did not modify the alpha diversity and the richness of the microbiota [80]. Furthermore, both study groups present a low alpha-diversity richness, a typical finding of subjects with AR, characterized by reduced alpha and beta diversity vs. healthy subjects [81].

The search for bacterial biomarkers revealed significant differences confirmed by changes in the relative abundance of a series of genera in both the Probiotic and Placebo groups. At T0, the Placebo group was positively associated with the *Ruminococcaceae UCG*-*014* genus, which is an intestinal butyrate producer [82], thus exerting a protective action against allergic symptoms [83]. This characteristic, however, was not maintained by the Placebo group across the three time points. At T2, the Probiotic group presented with a greater relative abundance of genera *Dorea* and *Fusicatenibacter* compared to the Placebo. Previous studies on subjects with AR observed a reduction in the abundance of the genus *Dorea*. This bacterium belongs to the phylum *Firmicutes* and the family Lachnospiraceae; in another study it was observed a reduction in the fecal levels of short-chain fatty acids of patients with AR that may be due to a reduction in *Dorea* [77,78,79,80,81]. This genus has been found to be reduced in children with food allergy or sensitization [84] or with AR [85] and in adult-onset atopic dermatitis (AOAD) [86] while *Fusicatenibacter* has anti-inflammatory properties, inducing IL-10 in intestinal mucosa to exert anti-inflammatory effects [87]. In children, the reduction in the abundance of *Fusicatenibacter saccharivorans* was associated with the future development of allergic disease, along with a reduction in butyric acid produced by this bacterium [88].

At T3, the Probiotic group showed a positive association and a higher abundance of *Streptococcus* compared to the Placebo group. This genus was found to be decreased in atopic dermatitis in infants [89]. *Streptococcus* and *Lactobacillus* can reduce sensitization to dust mites and belong to the Lactobacillales. A decreased abundance of this order has been observed in subjects sensitized to dust mites [90]. From a mechanistic standpoint, some strains of *Streptococcus* and *Lactobacillus* can stimulate Tregs of the lamina propria, resulting in a modulation of the Th1/Th2 balance [91]. Thus, the genus *Streptococcus* can play an anti-allergic role in the gut microbiota. Compared to the Probiotic group, at T3 the Placebo group showed a positive association and a higher relative abundance of *Bacteroides*, whose increase has been observed in asthma patients [92], as well as of *Ruminococcaceae unassigned Bacteroides*, which can be associated with allergic diseases [93]. Indeed, in children at three months of age, the increase in the *Bacteroides* genus and the reduction in *Bifidobacterium* are associated with a higher risk of developing allergic reactions at the age of 5 years [94]. High levels of *Bacteroides*, together with low levels of *Lactobacillus* and *Bifidobacterium*, are associated with the development of atopy; this is probably due to an excess of LPS that causes low-grade inflammation that mediates the Th2 response [95]. *Ruminococcus unassigned* is part of the intestinal commensals and their role in allergies is still unknown. At the end of the trial, the Placebo group maintained a low *Prevotella*/*Bacteroides* ratio, while the group treated with probiotics showed an increase in this ratio. Notably, in a pediatric study from China, the percentage of allergic disease, including AR, was higher in children living in an urban context than those who lived in a rural environment. From their analysis, it was observed that the *Prevotella*/*Bacteroides* ratio was higher in children coming from a rural context than in those from an urban context. Thus, the authors suggest that the high *Prevotella*/*Bacteroides* in subjects who lived in a rural context could exert a protective role against the development of allergic pathologies [96].

The capsule bulking agent contained maltodextrin, corn starch, and magnesium salt. The literature findings show that these components impact gut health and the microbiota differently. Maltodextrin, typically used as a food additive, appears to have a detrimental effect, contributing to intestinal inflammation [97]. In contrast, high-amylose corn starch, a form of resistant starch, effectively regulates gut microbiota and exhibits anti-obesity effects in mice made obese through a high-fat diet [98]. Magnesium’s impact on the microbiome is more significant. Recent studies have shown that fluctuations in dietary magnesium intake can directly influence gut microbiota in a time and dose-dependent manner [99]. To our knowledge, no studies have evaluated these components’ effects on AR. However, considering the modest quantity of these ingredients employed as bulking agents and the consistent use of the same agents in both the probiotic and placebo capsules, we conclude that their presence is unlikely to have compromised the study results.

The results of the present study identified a series of possible AR related bacteria (e.g., *Bacteroides* and *Ruminococcus*) that trigger low-grade inflammation via Th2-mediated response in the Placebo vs. Probiotic group biomarkers. Finally, the study identified possible biomarkers (e.g., *Dorea*, *Fusicatenibacter*, *Prevotella*) with anti-allergic and immunomodulatory properties that were more abundant in the Probiotic group than Placebo. This study involves a limited population sample (*n* = 41 subjects). Also, unsuccessful gut microbiota sequencing from two subjects in each of the placebo and probiotic groups has decreased the number of participants included in the NGS analysis to twenty in the placebo group and seventeen in the probiotic group. For this reason, the results should be interpreted considering that smaller samples, which diminishes statistical power, can lead to greater variability and may limit the ability to detect significant differences between the groups.

Thus, the limitations of the study are as follows: (i) the sample size was relatively small (*n* = 41), which may not be adequate to detect minor effects or to generalize the results to a larger population of individuals with AR; (ii) extended follow-up periods might be required for chronic conditions like AR to assess the long-term effects of probiotics; (iii) while this paper addresses changes in microbiota composition and related immune responses, it does not fully investigate the mechanisms through which probiotics produce anti-allergic effects. Therefore, future research would benefit from larger and more diverse participant cohorts to validate these results and clarify the underlying biological mechanisms. Additionally, the study used the MiniRQLQ to assess quality of life, which is a subjective evaluation that may be influenced by bias or individual differences in perception. Incorporating more objective measures of AR management in future studies could enhance the understanding of limitations.

## 5. Conclusions

Studies investigating the effects of probiotic interventions in AR patients on the gut microbiota composition and associated allergic symptoms are quite limited. Treatments targeted at rebalancing and preserving a healthy gut microbiota may be able to lessen the AR symptoms likely via immunocompetent mechanisms occurring in the intestine. In this clinical trial, the probiotic formulation containing 4 × 10^9^ UFC/capsule of *L. acidophilus* PBS066, *L. rhamnosus* LRH020, *B. breve* BB077, and *B. longum* subsp. *longum* BLG240 elicited anti-allergic and anti-inflammatory properties that were beneficial in improving symptoms and QoL of AR patients. As potential avenues for future research, it would be beneficial to explore in greater detail the underlying mechanisms of action of probiotics in the context of allergic rhinitis (AR) as well as to carry out studies that investigate larger sample sizes to enhance the reliability and validity of the findings.

## Data Availability

The original data presented in the study are openly available in the Sequence Read Archive (SRA) at https://www.ncbi.nlm.nih.gov/bioproject/1164129 (accessed on 24 September 2024) with project accession number PRJNA1164129.

## Supplementary Materials

The following supporting information can be downloaded at: https://www.mdpi.com/article/10.3390/nu16234173/s1. Supplementary Table S1. Inclusion and exclusion criteria applied to subjects of both sexes enrolled in the study. Figure S1: Violin plots of the alpha diversity analysis of the Placebo and Probiotic groups at the three time points considered (T0, T2, and T3). Analysis of the richness (Observed = Observed Species) diversity (Shannon = Shannon-Wiener index, and InvSimpson = Inverse Simpson’s index), and Phylogenetic diversity of the bacterial intestinal taxa. Median, first, and third quartiles are shown in the violin plots. Mann–Whitney U Test was used to perform group comparisons; Figure S2: Beta Diversity. Unweighted and weighted UniFrac PCoA (Principal component analysis) of intestinal bacterial taxa in the Placebo and Probiotic group at the three time points considered (T0, T2, and T3). Every dot represents a sample of the study. The horizontal axes (Axis 2 and Axis 3, and Axis 1 and Axis 2, respectively) explained 6.3% and 4,1% of the variation in the unweighted UniFrac PCofA, and the 22.3% and 12.9% of the variation in the weighted UniFrac PCoA, respectively; Figure S3: Mean relative abundance (%) of bacterial phyla in the Placebo and Probiotic groups at the three time points considered in our study (T0, T2, and T3). Figure S4. (a) eosinophil count, (b) eosinophil cationic protein (ECP), and (c) Total IgE (tIgE) at T0, T2, T3 in Probiotic and Placebo group. No statistically significant intragroup and intergroup differences were found (*p* > 0.05) [100,101].
