# Supplementary material for: Clinical Efficacy of Probiotics for Allergic Rhinitis: Results of an Exploratory Randomized Controlled Trial
Source: Nutrients. 2024 Nov 30;16(23):4173. doi: 10.3390/nu16234173 (PMC11644003; doi:10.3390/nu16234173)
Supplement: Supplementary file 1 [file nutrients-16-04173-s001.zip › nutrients-3317615-supplementary.pdf]

Supplementary data

1. Inclusion and exclusion criteria applied to the subjects of both sexes enrolled in the study.

**Inclusion criteria were:**
symptoms of seasonal or persistent AR (in the case of seasonal rhinitis, they were enrolled before and during pre-seasonal pollen);
willingness and ability to participate in the study;
willingness not to change their normal daily routine (lifestyle, physical activities, etc.) throughout the duration of the study;
willingness not to change their normal diet for the entire duration of the study;
willingness to use only the product to be tested for the entire duration of the study;
willingness not to use similar products for the entire duration of the study;
willingness not to use products that may interfere with the product to be tested;
willingness not to participate in similar studies;
signed the informed consent and be aware of the procedures of the study.
**Exclusion criteria were:**
subjects who did not respect the inclusion criteria;
subjects with suspected or confirmed sensitivity to one or more components of the product;
subjects with a clear history of chronic diseases (congenital cardiovascular diseases, diseases of the liver and kidney, or immunodeficiency);
subjects undergoing pharmacological and/or antibiotic treatment (currently);
subjects who cannot stop drugs that can affect rhinitis symptoms during the period of the study;

subjects who had other ongoing concomitant pathologies (immune, infectious, respiratory, or gastro-intestinal);
subjects with uncontrolled asthma;
subjects undergoing treatment with systemic steroids within 2 weeks of the start of the study;
subjects with gastritis and undergoing treatment with a proton pump inhibitor or anti-H2 receptor antagonists;
subjects recently (less than 3 months) subjected to immunosuppressant therapy;
subjects with ongoing serious illnesses;
subjects using illicit drugs and/or with alcohol abuse;
subjects who were deemed by the investigator to be unsuitable for participation for any reason;
subjects who were unable to communicate or cooperate with the physicians involved in the trial due to speech problem, mental retardation, or impaired brain function.

Supplementary Table 1. Inclusion and exclusion criteria applied to subjects of both sexes enrolled in the study.

2. Alpha diversity

Microbial alpha diversity was assessed using Observed Species, Shannon-Wiener, and Inverse Simpson's index. The Observed Species index estimates the number of different species in a community. The Shannon–Wiener index and the Inverse Simpson's index evaluate both richness and evenness. Anyway, the Shannon–Wiener index emphasizes mainly rare taxa, while the Inverse Simpson's index assigns more weight to dominant microorganisms [100]. Phylogenetic diversity (PD) describes the biodiversity of bacterial taxa based on the branch lengths of the phylogenetic tree, thus evaluates the relationship among bacterial microorganisms in the community: the higher the PD, the more distantly related the community elements are. All sequenced samples showed no significant changes in the richness (Observed Species), bacterial taxa diversity (Shannon-Wiener index and Inverse Simpson's index), or phylogenetic diversity in the intestinal ecosystem during and after the treatments.

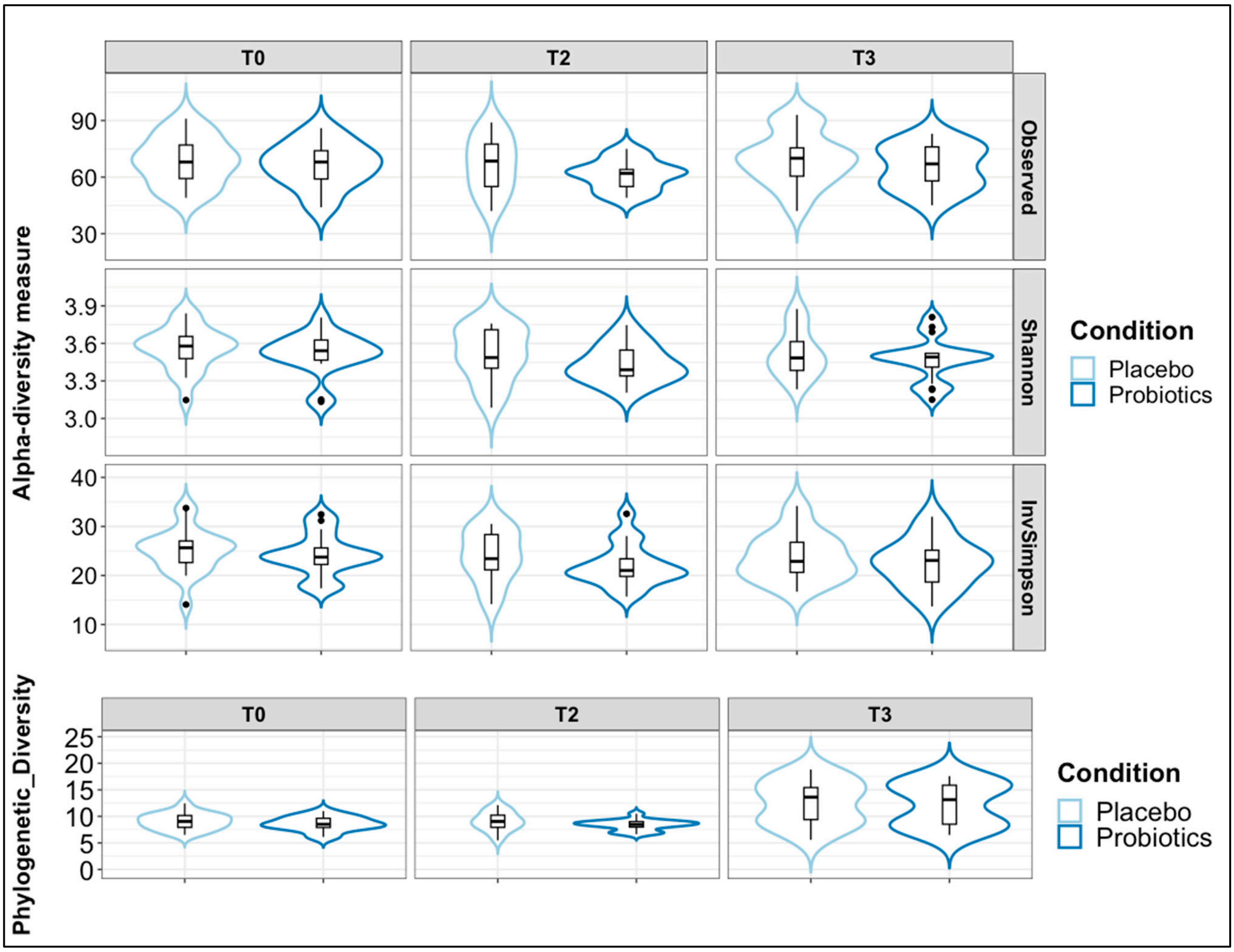

Supplementary Figure S1. Violin plots of the alpha diversity analysis of the Placebo and Probiotic groups at the three time points considered (T0, T2, and T3). Analysis of the richness (Observed = Observed Species), diversity (Shannon = Shannon-Wiener index, and InvSimpson = Inverse Simpson's index) and Phylogenetic diversity of the bacterial intestinal taxa. Median, first, and third quartiles are shown in the violin plots. Mann–Whitney *U* Test was used for group comparisons.

3. Beta diversity

The beta diversity was evaluated using the unweighted and weighted UniFrac distance metric. The former uses the taxa presence or absence in the sample, while the latter weights the branch length with the taxa relative abundance. However, both methods consider the phylogenetic distance of the taxa in the samples [101]. The unweighted and weighted UniFrac analysis did not show significant differences in the distribution of bacterial taxa between the two groups (Placebo and Probiotic) at the three time points investigated (PERMANOVA [Pr(>F)] > 0.05).

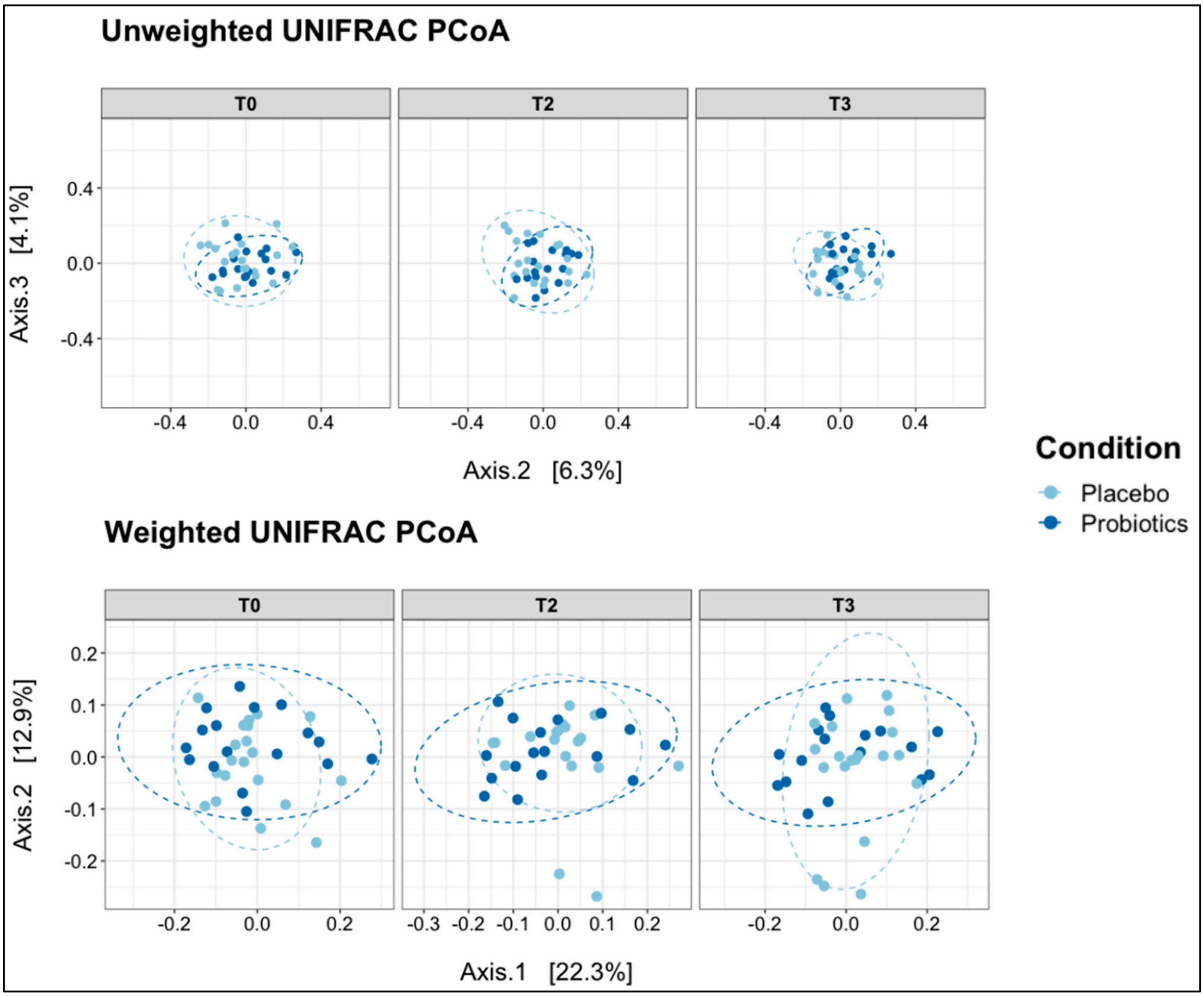

Supplementary Figure S2. Beta Diversity. Unweighted and weighted UniFrac PCoA (Principal component analysis) of intestinal bacterial taxa in the Placebo and Probiotic group at the three time points considered (T0, T2, and T3). Every dot represents a sample of the study. The horizontal axes (Axis 2 and Axis 3, and Axis 1 and Axis 2, respectively) explained 6.3% and 4,1% of the variation in the unweighted UniFrac PCoA, and the 22.3% and 12.9% of the variation in the weighted UniFrac PCoA, respectively.

4. Relative abundance of phyla

The mean relative abundance of the bacterial phyla remained similar at each time point within both groups. *Bacteroidetes* and *Firmicutes* were the most highly represented phyla in all samples.

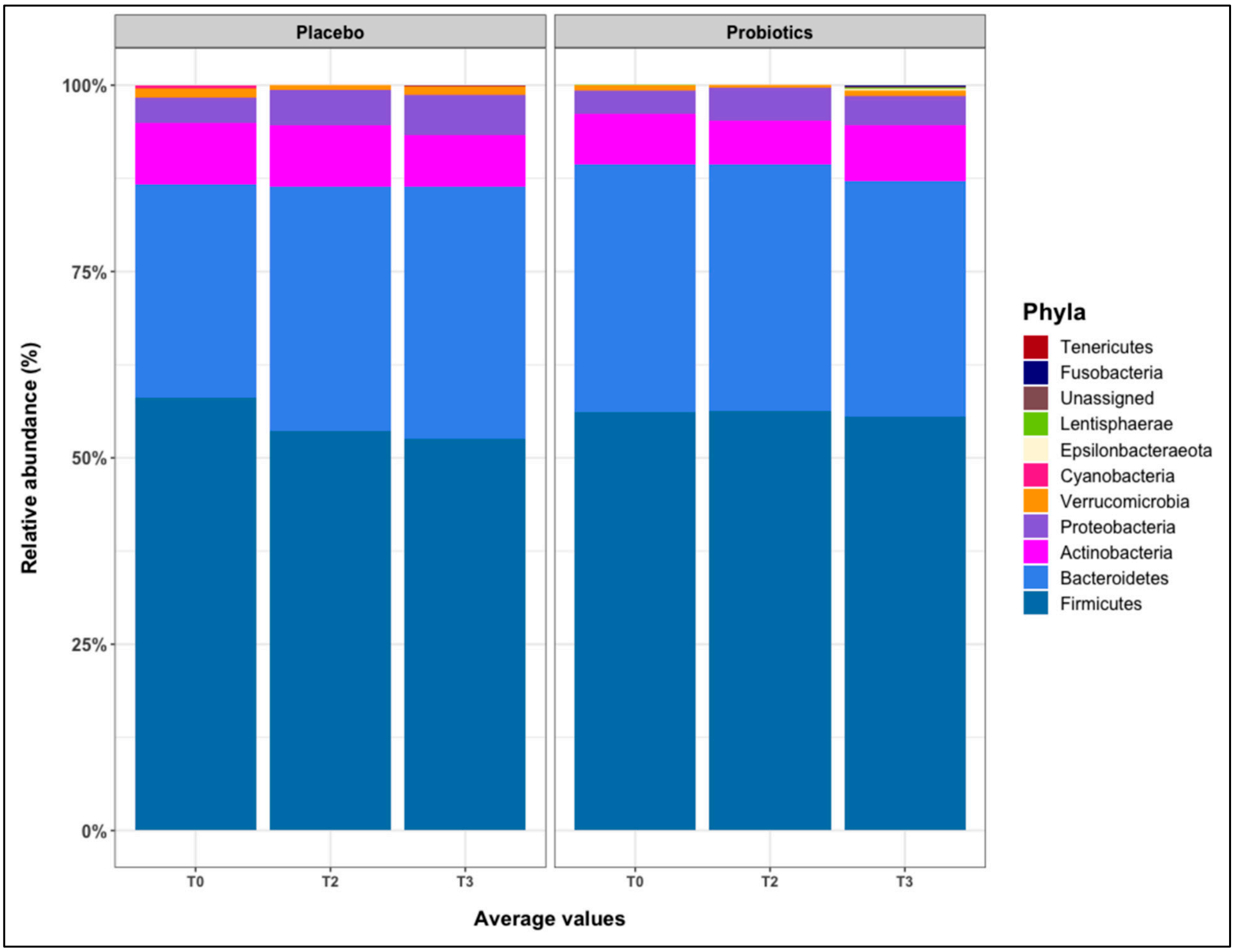

Supplementary Figure S3. Mean relative abundance (%) of bacterial phyla in the Placebo and Probiotics groups at the three time points considered in our study (T0, T2, and T3).

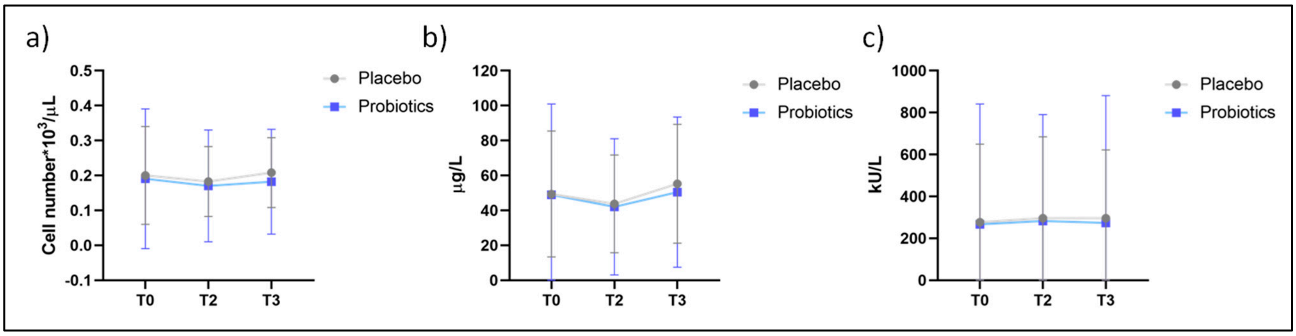

Supplementary Figure S4. a) eosinophil count, b) eosinophil cationic protein (ECP), and c) Total IgE (tIgE) at T0,T2,T3 in Probiotic and Placebo group. No statistically significant intragroup and intergroup differences were found ($p>0.05$).
